# Differential miRNA expression of hypoxic MCF7 and PANC-1 cells

**DOI:** 10.3389/fendo.2023.1110743

**Published:** 2023-07-31

**Authors:** Sandy M. Al-Sisan, Malek A. Zihlif, Hana M. Hammad

**Affiliations:** ^1^ Department of Pharmacology, School of Medicine, The University of Jordan, Amman, Jordan; ^2^ Department of Biological Sciences, School of Science, The University of Jordan, Amman, Jordan

**Keywords:** breast cancer, cyclin dependent kinase, epithelial-to-mesenchymal transition, miRNA, pancreatic ductal adenocarcinoma, chronic cyclic hypoxia, hypoxamirs, doxorubicin chemoresistance

## Abstract

**Background:**

Hypoxia plays a critical role in the tumor microenvironment by affecting cellular proliferation, metabolism, apoptosis, DNA repair, and chemoresistance. Since hypoxia provokes a distinct shift of microRNA, it is important to illustrate the relative contribution of each hypoxamiR to cancer progression.

**Aims:**

The present study aims to shed light on the hypoxamiRs that are involved in pancreatic and breast cancer progression to highlight novel targets for the development of new therapies.

**Methods:**

For 20 cycles, MCF7 breast cancer cells and PANC-1 pancreatic cancer cells were subjected to chronic cyclic hypoxia, which consisted of 72 hours of hypoxia followed by 24 hours of reoxygenation. After 10 and 20 cycles of hypoxia, miRNA expression alterations were profiled using RT-PCR array and further analyzed using a visual analytics platform. The MTT cell proliferation assay was used to determine hypoxic cells’ chemoresistance to doxorubicin.

**Results:**

Under chronic cyclic hypoxia, hypoxic PANC-1 cells have a comparable doubling time with their normoxic counterparts, whereas hypoxic MCF7 cells show a massive increase in doubling time when compared to their normoxic counterparts. Both hypoxic cell lines developed EMT-like phenotypes as well as doxorubicin resistance. According to the findings of miRNet, 6 and 10 miRNAs were shown to play an important role in enriching six hallmarks of pancreatic cancer in the 10th and 20th cycles of hypoxia, respectively, while 7 and 11 miRNAs were shown to play an important role in enriching the four hallmarks of breast cancer in the 10th and 20th cycles of hypoxia, respectively.

**Conclusions:**

miR-221, miR-21, miR-155, and miR-34 were found to be involved in the potentiation of hypoxic PANC-1 hallmarks at both the 10th and 20th cycles, while miR-93, miR-20a, miR-15, and miR-17 were found to be involved in the potentiation of hypoxic MCF7 hallmarks at both the 10th and 20th cycles. This variation in miRNA expression was also connected to the emergence of an EMT-like phenotype, alterations in proliferation rates, and doxorubicin resistance. The chemosensitivity results revealed that chronic cyclic hypoxia is critical in the formation of chemoresistant phenotypes in pancreatic and breast cancer cells. miR-181a and let-7e expression disparities in PANC1, as well as miR-93, miR-34, and miR-27 expression disparities in MCF7, may be associated with the formation of chemoresistant MCF7 and PANC-1 cells following 20 cycles of chronic cyclic hypoxia. Indeed, further research is needed since the particular mechanisms that govern these processes are unknown.

## 1 Introduction

Cancer is the devastating growth of cells along with inhibition of apoptotic pathways as well as the acquisition of metastatic characteristics via triggering oncogenes or frustrating tumor suppressor genes. Breast cancer (BC) ranks as the most common cancer in women. Many cases of BC have shown dissimilar clinical results despite having similar characteristics. In addition to drug resistance cases that arise as a result of complexity, heterogeneity, and metastasis (1), the level of hypoxia (oxygen deprivation) within the tumor microenvironment is also a factor that complicates tumor progression, metastasis, and the survival of breast cancer patients (2).

On the other hand, pancreatic ductal adenocarcinoma (PDAC) is one of the most fatal cancers worldwide, with a mortality rate almost equal to the incidence rate (3). The absence of early worrisome symptoms, rapid metastasis, highly malignant patterns, and an innate resistance to conventional chemotherapy treatments are the primary causes of PDAC prognosis (4). Furthermore, PDAC possesses therapeutic-obstructive features such as heterogeneity, a desmoplastic and hypoxic tumor microenvironment (TME), intrinsic resistance to chemotherapy, and immunotherapy resistance (5). The limitations of current treatment strategies for breast cancer and PDAC emphasize the pressing need to explore key molecular pathways related to early diagnosis, prognosis, and therapy improvement.

Cancer patients rarely die from primary tumors; cancer cells’ metastatic capacity is responsible for the severity, aggressiveness, and majority of cancer-related deaths. Hypoxia performs a critical function in triggering metastasis through manipulation of the tumor microenvironment (TME). It also promotes drug resistance, angiogenesis, metabolic reprogramming, and proliferation by interfering with different regulatory networks of transcription factors and signaling proteins. Accordingly, hypoxia can boost several hallmarks of cancer (6). Recent studies demonstrated that microRNA plays an important role in the adaptive response to hypoxia in tumors. Modification of specific miRNAs, collectively called hypoxamiRs, has become a remarkable feature of hypoxia (7). HypoxamiRs are microRNAs that are not only tightly regulated by hypoxia but also influence cell responses to reduced oxygen tension (8). A growing body of research suggests that hypoxia controls several aspects of hypoxamiR transcription and function (9). Thus, it is essential to define the distinct expression of miRNAs in different types of cancers to suggest specific treatment strategies and avoid any off-target effects.

Our goal was to figure out how miRNAs interact with the hypoxic microenvironment and what function this interaction plays in carcinogenesis. The hypoxic microenvironment of cancer cells is simulated, as closely as feasible, by exposing breast and pancreatic cancer cells to chronic cyclic hypoxia for 20 cycles. This enables the detection of changes in miRNA expression patterns and their consequences for cellular regulatory processes, possibly leading to the identification of novel therapeutic targets.

## 2 Materials and methods

### 2.1 Cell culture

Pancreatic cancer cell line (PANC-1) and breast adenocarcinoma cell line (MCF7) were purchased from the American Type Culture Collection (ATCC, USA). PANC-1 cells were cultured in Dulbecco’s Modified Medium High Glucose (Euro clone, Italy) while MCF7 cells were cultured in RPMI 1640 medium (Euro clone, Italy), each of which was supplemented with 10% (v/v) heat-inactivated fetal bovine serum (Biowest, South America), 1% of 2mM L-glutamine (Euro Clone, Italy), and 100U/ml and 100µg/ml penicillin-streptomycin (Euro Clone, Italy). Cells were routinely propagated and cultured in a cell culture incubator (Nuaire, USA) at 37°C and (5% CO_2_) 95% atmospheric air (normoxic conditions); then each cell line was divided into non-hypoxic and hypoxic cells as a control and experimental group, respectively.

### 2.2 Cells passaging

When the confluence of adherent cells reached 80%, the cells were passaged using Trypsin-EDTA (0.05%) in DPBS (1x) (Capricon Scientific, Germany) to allow them to survive, grow and divide. PANC-1 and MCF7 cancer cells were counted and evaluated in terms of viability via trypan blue 0.5% solution (Biowest, South America); after 1:1 dilution of homogenized trypan blue and cells, the solution was loaded onto a hemacytometer (Neubauer Double, Zuzi, Spain) and thus could be counted. The doubling time of both the control and hypoxic cells was calculated and compared throughout the experiment.

### 2.3 Exposure to hypoxia

AnaeroGen Compact (Oxoid; Hampshire, UK) was used for hypoxic episode application. The system encompasses a tightly sealed bag with a gas-generating sachet. Each sachet contains activated carbon and ascorbic acid, which combine with the air when opened, consuming oxygen and reducing its concentration within the bag to less than 1%. Culture flasks and opened sachets were placed inside plastic pouches, which were then sealed. PANC-1 and MCF7 cells were exposed to a 72-hour hypoxic cycle using the abovementioned system followed by 24-48 hours of reoxygenation. Cells were subjected to 20 cycles of hypoxia. Apart from these signified cycles, control cells were split beside their hypoxic counterparts and were subjected to similar conditions except hypoxic cycles (10, 11).

### 2.4 Cell proliferation assay

PANC-1 and MCF7 viability, for both hypoxic and normoxic cells, was evaluated after the10^th^ and 20^th^ hypoxic cycles using a Cell Titer 96^®^ Non-Radioactive Cell Proliferation Assay (MTT) (Promega, USA). Doxorubicin 50mg/25 ml (Ebewe, Austria) was used in descending concentrations, from 25µM to 0.1 µM, to assess the chemo-sensitivity of both groups. Thereafter, optical density was determined using a Synergy multi-mode reader (Biotek, USA) at a wavelength of 570 nm; consequently, the cells’ viability (%) could be compared. Statistical analysis numerical data were expressed as mean ± SD and the statistical significance was determined by using the Student’s t-test.

### 2.5 Total ribonucleic acid extraction

After the 10^th^ and 20^th^ hypoxic cycles, 3×10^3^ cells of each cell line (PANC-1 and MCF7) were subjected to total RNA extraction according to the miRNeasy Mini Kit (Qiagen, Germany) quick start protocol; RNA samples were preserved at -80°C for the next step. RNA purity and concentrations of RNA samples were assessed using a NanoDrop 2000 UV-vis spectrophotometer (Thermo Scientific, USA). Each concentration was tested in duplicate.

### 2.6 miRNA expression profiling

The extracted total RNA was converted to complementary DNA (cDNA) using the miScript ll RT kit (Qiagen, SABIO). The miScript SYBR^®^ Green PCR ready master mix was used to prepare samples, and levels of expression of human breast cancer and pancreatic cancer miRNAs were investigated using a Human Hypoxia Signaling miScript miRNA PCR Array (Qiagen, SABIO). The panels of miRNA that were used in this research were ready-to-use PCR panels (Qiagen, SABIO), with a 96-well format that contained 84 miRNA assays targeting human hypoxia signaling, reference miRNAs, and control assays, according to the manufacturer’s instructions, via a Bio-Rad CFX96 instrument (Qiagen, Germany). Variation in the cDNA content was normalized using different small nuclear and small nucleolar RNAs (snRNAs and snoRNAs) like SNORD61, SNORD68, SNORD72, SNORD95, SNORD96A, and RNU6-6P (Appendix D). Reverse transcription control and positive PCR control were also performed. The 2^-ΔΔCt^ method was used to calculate the relative expression levels of the miRNA. miRNAs expression analysis was performed using the GeneGlobe Data Analysis Center from Qiagen. The arrays’ reproducibility were determined by analyzing two samples from each group in duplicate.

### 2.7 miRNA pathway-enriched analysis

miRNAs with more than twofold changes in expression levels were further analyzed and classified using a miRNA-centric network visual analytics platform (miRNet). Based on the IMEx interactome, literature-curated comprehensive data from InnateDB (12), we highlighted the miRNAs that were exclusively related to our investigation (miRNAs related to breast cancer and miRNAs related to pancreatic cancer), and then the miRNAs were filtered to distinguish miRNAs that are highly related to different cancer pathways with a p-value less than 0.05.

The p-value is primarily dependent on network connectivity and indicates how significant the connections within a specified miRNA function are. The edges of a certain function were considered “internal,” and the edges connecting the nodes of that function to the rest of the graph were considered “external.” The test’s null hypothesis was that there is no difference in the number of “internal” and “external” connections to a given node in the miRNA function. A Wilcoxon rank-sum test of the “internal” and “external” degrees was used to compute the p-value.

The degree of a node in a graph network is the number of connections it has to other nodes. In a network, nodes having a higher node degree function as hubs. Nodes having a degree of 100 or higher were selected in this study.

## 3 Results

### 3.1 Total RNA quality and quantity

At cycles 10 and 20, RNA was extracted from hypoxic PANC-1 and MCF7 cells, as well as their normoxic counterparts (see **Table 1 in the Supplementary Material**).

### 3.2 Resistance to doxorubicin

Resistance to the commonly used chemotherapeutic agent, doxorubicin, was examined to ensure the development of the hypoxic phenotype and to understand the effect of hypoxia on doxorubicin resistance after 10 and 20 cycles of exposure to hypoxic conditions. For both hypoxic and normoxic cancer cells, each doxorubicin concentration was tested in triplicate.

#### 3.2.1 PANC-1 doxorubicin resistance

**Figure 1:**
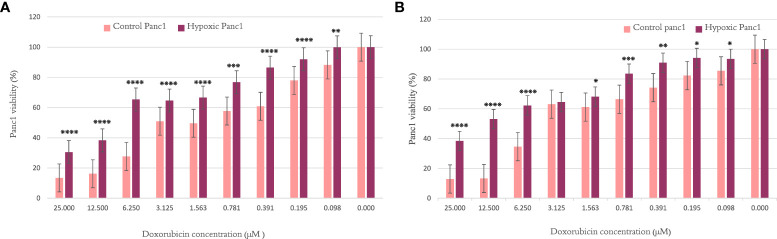
The effect of 10 and 20 cycles of chronic cyclic hypoxia on doxorubicin-induced cytotoxicity of PANC-1. **(A)** Represents the effect after 10 cycles, **(B)** represents the effect after 20 cycles. Statistical significance was determined by using the Student’s t-test.*p-value <0.05, **p-value <0.01, ***p-value <0.001, ****p-value <0.0001.

When investigating the chemosensitivity of PANC-1 after cycles 10 and 20, the findings in **Figures 1A, B** show that the PANC-1 viability percentage was significantly higher for hypoxic cells than their normoxic counterparts per concentration of doxorubicin. The results are illustrated as mean ± SEM, and the p-values were determined using the unpaired t-test.

#### 3.2.2 MCF7 doxorubicin resistance

**Figure 2:**
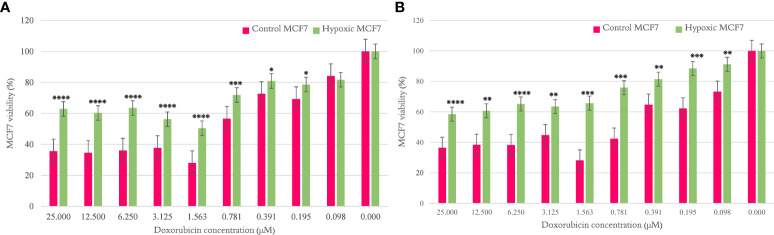
The effect of 10 and 20 cycles of chronic cyclic hypoxia on doxorubicin-induced cytotoxicity of MCF7. **(A)** Represents the effect after 10 cycles, **(B)** represents the effect after 20 cycles. Statistical significance was determined by using the Student’s t-test.*p-value <0.05, **p-value <0.01, ***p-value <0.001, ****p-value <0.0001.

The MCF7 viability percentage was significantly higher for hypoxic cells than normal counterparts per concentration of doxorubicin, as shown in **Figures 2A, B**, when examined after cycles 10 and 20, respectively. The results are illustrated as mean ± SEM, and the p-values were determined using the unpaired t-test.

### 3.3 Differential miRNA expression

In the present study, we examined miRNA expression changes after 10 and 20 cycles of chronic cyclic hypoxia, 1% O_2_, 72 hours of hypoxia, and 24 hours of reoxygenation (H-R: 72-24), in pancreatic and breast cancer cell lines. A standard twofold expression change was used as an arbitrary cutoff. The human hypoxia signaling miRNA PCR array revealed several deregulated miRNAs in hypoxic cells when compared with their normoxic counterparts.

#### 3.3.1 Changes in PANC-1 miRNA expression pattern

When PANC-1 cells exposed to 10 and 20 cycles of hypoxia were compared with their normoxic counterparts, expression analysis revealed significant changes in 25 miRNA at the 10th cycle. The expression of five of these miRNAs were significantly upregulated, while those of 20 miRNAs were significantly downregulated. At the 20th cycle, significant changes in 24 miRNAs were revealed. The expressions of six of these miRNAs were significantly upregulated, while those of 18 miRNAs were significantly downregulated. The PCR array-based miRNA transcriptomic profiling of PANC-1 is presented in more detail in **Supplementary Tables 2**, **3**.

#### 3.3.2 Changes in MCF7 miRNA expression pattern

When MCF7 cells exposed to 10 and 20 cycles of hypoxia were compared with their normoxic counterparts, miRNA expression analysis revealed significant changes in 19 miRNA at the 10th cycle. The expressions of six of these miRNAs were significantly upregulated, while those of 13 miRNAs were significantly downregulated. At the 20th cycle, significant changes in 47 miRNAs were revealed. The expression of two of these miRNAs were significantly upregulated, while those of 45 miRNAs were significantly downregulated. The PCR array-based miRNA transcriptomic profiling of MCF7 is presented in more detail in **Supplementary Tables 4, 5**.

### 3.4 Interaction network analysis (miRNet)

We constructed the miRNA gene network based on specific miRNA–disease relationships to classify the functional units and putative enriched pathways in which the miRNAs are involved.

#### 3.4.1 Enriched pathway of hypoxic PANC-1

Many miRNAs with high levels of cancer pathways enrichment in pancreatic cancer were detected by the network, including let-7f and miR-15b at the 10^th^ cycle. The 20^th^ cycle included miR-135, miR-101, miR-7, miR-378, miR-181a, and miR-29, as well as miR-155, miR-21, miR-221, and miR-34a, which were involved in both cycles. The results are given in **Tables 1**, **2** for the 10^th^ and 20^th^ cycles, respectively. The p-values were primarily dependent on network connectivity and indicate how significant the connections within a specified miRNA function are.

**Table 1:** miRNet analysis of PANC-1 key pathways after 10 hypoxic cycles.

Cancer hallmark	miRNA function	P-value	miRNA	Fold change
Activation invasion and metastasis	Wound healing	0.00534	hsa-miR-21-5p	-2.366
			hsa-miR-34a-5p	-3.607
			hsa-miR-155-5p	-3.0619
Inducing angiogenesis	Angiogenesis	0.0115	hsa-let-7f-5p	-4.0676
			hsa-miR-21-5p	-2.366
			hsa-miR-34a-5p	4.5799
Tumor promoting inflammation	Inflammation	0.013	hsa-let-7f-5p	-4.0676
			hsa-miR-34a-5p	4.5799
			hsa-miR-221-3p	-2.389
			hsa-miR-155-5p	-3.0619
Sustaining proliferative signals	Cell cycle	0.0133	hsa-miR-21-5p	-2.366
			hsa-miR-34a-5p	4.5799
			hsa-miR-221-3p	-2.389
			hsa-miR-15b-5p	-3.607
	Cell proliferation	0.0141	hsa-miR-21-5p	-2.366
			hsa-miR-34a-5p	4.5799
			hsa-miR-221-3p	-2.389
			hsa-miR-15b-5p	-3.607
Resisting cell death	Cell death	0.0153	hsa-let-7f-5p	-4.0676
			hsa-miR-21-5p	-2.366
			hsa-miR-34a-5p	4.5799
			hsa-miR-221-3p	-2.389
Genome instability and mutation	DNA damage response	0.0332	hsa-miR-34a-5p	4.5799
			hsa-miR-15b-5p	-3.607
	DNA damage repair	0.04	hsa-miR-34a-5p	4.5799
			hsa-miR-155-5p	-3.0619

**Table 2:** miRNet analysis of PANC-1 key pathways after 20 hypoxic cycles.

Cancer hallmark	miRNA function	P-value	miRNA	Fold change
Activation invasion and metastasis	Wound healing	0.00044	hsa-miR-21-5p	-2.1917
			hsa-miR-29b-3p	-2.3059
			hsa-miR-34a-5p	6.0687
			hsa-miR-155-5p	-4.1415
			hsa-miR-378a-3p	-2.7188
Tumor promoting inflammation	Inflammation	0.00121	hsa-miR-101-3p	-3.4783
			hsa-miR-7-5p	-5.717
			hsa-miR-34a-5p	6.0687
			hsa-miR-181a-5p	2.9116
			hsa-miR-221-3p	-2.0319
			hsa-miR-135a-5p	-3.4289
			hsa-miR-155-5p	-4.1415
Resisting cell death	Apoptosis	0.0013	hsa-miR-101-3p	-3.4783
			hsa-miR-29b-3p	-2.3059
			hsa-miR-7-5p	-5.717
			hsa-miR-34a-5p	6.0687
			hsa-miR-181a-5p	2.9116
			hsa-miR-135a-5p	-3.4289
			hsa-miR-155-5p	-4.1415
	Cell death	0.00999	hsa-miR-21-5p	-2.1917
			hsa-miR-29b-3p	-2.3059
			hsa-miR-7-5p	-5.717
			hsa-miR-34a-5p	6.0687
			hsa-miR-181a-5p	2.9116
			hsa-miR-221-3p	-2.0319
Genome instability and mutation	DNA damage repair	0.0199	hsa-miR-101-3p	-3.4783
			hsa-miR-34a-5p	6.0687
			hsa-miR-155-5p	-4.1415
Sustaining proliferative signals	Regulation of Akt pathway	0.0497	hsa-miR-7-5p	-5.717
			hsa-miR-221-3p	-2.0319
			hsa-miR-155-5p	-4.1415
	Cell proliferation	0.0415	hsa-miR-21-5p	-2.1917
			hsa-miR-29b-3p	-2.3059
			hsa-miR-34a-5p	6.0687
			hsa-miR-221-3p	-2.0319
			hsa-miR-378a-3p	-2.1917

#### 3.4.2 Enriched pathway of hypoxic MCF7

At the 10th cycle, the network identified several miRNA molecules with a high degree of hallmarks enrichment in breast cancer, including miR-27, miR-34, and miR-205. In the 20th cycle, miR-15b, miR-16, miR-7, miR-101, miR-9, and let-7a all played important roles. The two cycles both identified miR-93, miR-15a, miR-17, and miR-20a. **Tables 3**, **4** encompass the results for the 10^th^ and 20^th^ cycles, respectively. The p-values were primarily dependent on network connectivity and indicate how significant the connections within a specified miRNA function are.

**Table 3:** miRNet analysis of MCF7 key pathways after 10 hypoxic cycles.

Cancer hallmark	miRNA function	P-value	miRNA	Fold change
Inducing angiogenesis	Angiogenesis	0.000656	hsa-miR-15a-5p	-3.3759
			hsa-miR-17-5p	-2.6435
			hsa-miR-20a-5p	-2.5648
			hsa-miR-93-5p	-2.4855
			hsa-miR-34a-5p	2.3543
			hsa-miR-205-5p	-3.3347
Genome instability and mutation	DNA damage response	0.00574	hsa-miR-15a-5p	-3.3759
			hsa-miR-27a-3p	2.3356
	Cell proliferation	0.00396	hsa-miR-34a-5p	2.3543
Sustaining proliferative signals			hsa-miR-17-5p	-2.6435
			hsa-miR-20a-5p	-2.5648
	Cell division	0.00645	hsa-miR-15a-5p	-3.3759
			hsa-miR-27a-3p	2.3356
			hsa-miR-34a-5p	2.3543
	Regulation of Akt pathway	0.0201	hsa-miR-17-5p	-2.6435
			hsa-miR-20a-5p	-2.5648
			hsa-miR-205-5p	-3.3347

**Table 4:** miRNet analysis of MCF7 key pathways after 20 hypoxic cycles.

Cancer hallmark	miRNA function	P-value	miRNA	Fold change
Sustaining proliferative signals	Cell proliferation	0.00908	hsa-miR-17-5p	-5.0454
			hsa-miR-20a-5p	-5.7934
	Cell cycle	0.00253	hsa-let-7a-5p	-2.2752
			hsa-miR-16-5p	-5.3026
			hsa-miR-17-5p	-5.0454
			hsa-miR-20a-5p	-5.7934
			hsa-miR-15b-5p	-2.3688
			hsa-miR-9-5p	-2.7556
			hsa-miR-195-5p	-6.1639
Resisting cell death	Apoptosis	0.0143	hsa-miR-15a-5p	-2.3688
			hsa-miR-16-5p	-5.3026
			hsa-miR-101-3p	-4.9598
			hsa-miR-7-5p	-2.0717
			hsa-miR-9-5p	-2.7556
			hsa-miR-195-5p	-6.1639
Genome instability and mutation	DNA damage response	0.0195	hsa-miR-15a-5p	-2.3688
			hsa-miR-16-5p	-5.3026
			hsa-miR-15b-5p	-2.3688
Inducing angiogenesis	Angiogenesis	0.0486	hsa-miR-15a-5p	-2.3688
			hsa-miR-16-5p	-5.3026
			hsa-miR-17-5p	-5.0454
			hsa-miR-20a-5p	-5.7934
			hsa-miR-93-5p	-4.6331

#### 3.4.3 The co-enrichment pathways between hypoxic MCF7 and hypoxic PANC1

Sustaining proliferative signals and resisting cell death were both enriched in PANC1 and MCF7 hypoxic cells, as indicated in **Table 5**.

**Table 5:** The hypoxic MCF7 and hypoxic PANC1 co-enrichment pathways.

Cancer hallmark	Cycle number	miRNA symbol	MCF7 fold change	PANC-1 fold change
Sustaining proliferative signals	10	hsa-miR-34a-5p	2.3543	4.5799
Resisting cell death	20	hsa-miR-101-3p	-4.9598	-3.4783
		hsa-miR-7-5p	-2.0717	-5.717

### 3.5 Phenotypic alteration under chronic cyclic hypoxia

We merely describe a change in the morphology of cells that acquire an elongated shape and lose their polarity when observed under the microscope, but further investigation into epithelial-to-mesenchymal transition (EMT) biomarkers is necessary to confirm this change.

#### 3.5.1 PANC-1 phenotypic switch

**Figure 3:**
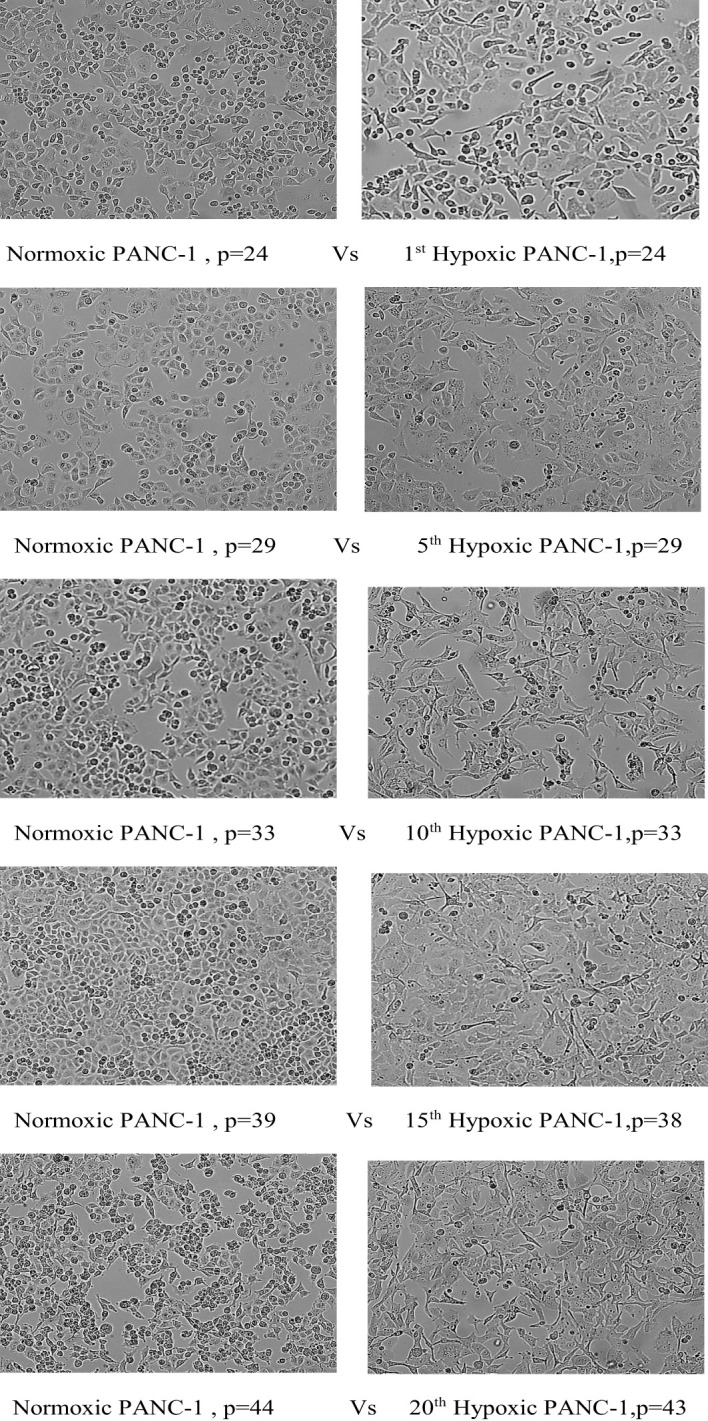
Morphological change of PANC-1 cell lines under chronic cyclic hypoxia. Normoxic PANC-1 vs. hypoxic PANC-1, (10x magnification). P: passage number.

The morphological switch from epithelial to mesenchymal was observed throughout the hypoxia cycles, starting obviously with the fifth cycle. PANC-1 maintained a comparable proliferation rate throughout the experiment for hypoxic and normal cells, beginning with a 43-hour doubling time for both, a 28-hour doubling time for both at cycle 10, and a 22-hour doubling time for normoxic PANC-1 at cycle 20, while the doubling time for hypoxic cells remained unchanged from cycle 10. PANC-1 cells underwent morphological changes after 5, 10, 15, and 20 cycles of hypoxia, as seen in **Figure 3**.

#### 3.5.2 MCF7 phenotypic switch

**Figure 4:**
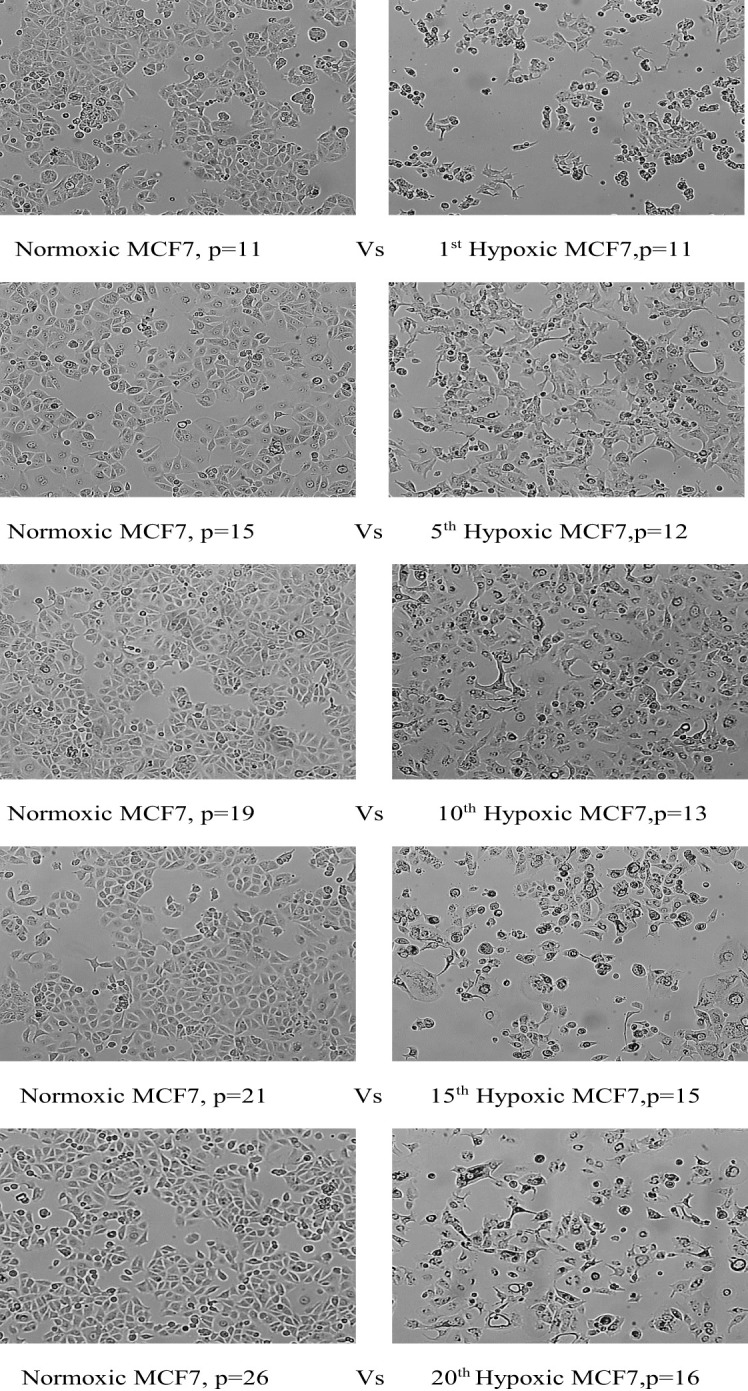
Morphological change of MCF7 cell lines under chronic cyclic hypoxia. Normoxic MCF7 vs. hypoxic MCF7, (10x magnification). P: passage number.

Hypoxic MCF7 cells proliferated at a slower rate than their normal counterparts; the doubling time of hypoxic cells was greatly increased to 84 hours in the 10th cycle, compared with 42 hours for normoxia, and in cycle 20, normal cells rapidly proliferated with a doubling time of 33 hours while the doubling time for hypoxic cells was further increased to 89 hours. However, hypoxic MCF7 cell proliferation was significantly reduced at cycle 12, allowing us to expand the reoxygenation period to 5 days between cycles 12 and 15. In terms of EMT development, MCF7 lost cell–cell adhesion during all hypoxic cycles, gaining an elongated shape and losing its polarization, as seen in **Figure 4**.

## 4 Discussion

To uncover novel hypoxamiR targets in cancer, we exposed MCF7 breast cancer cells and PANC-1 pancreatic cancer cells to 20 cycles of chronic cyclic hypoxia (72 h H, 24 h R). The MTT proliferation assay was used to determine resistance to doxorubicin after 10 and 20 cycles. In addition, miRNA expression profiling was performed mid-experiment (after 10 cycles of hypoxia) and at completion (after 20 cycles of hypoxia). According to the findings, chronic cyclic hypoxia had a key role in the formation of a chemoresistant phenotype in the MCF7 breast cancer cell line and the PANC-1 pancreatic cancer cell line. Furthermore, cells subjected to 20 cycles of chronic cyclic hypoxia showed substantial changes in miRNA expression profile when compared with their normoxic counterparts. Likewise, significant alterations were seen in cells subjected to 10 cycles, sharing several characteristics with those exposed to 20 cycles. We were able to induce doxorubicin resistance in MCF7 and PANC-1 cells after exposing them to 10 and 20 cycles of chronic cyclic hypoxia. This was verified by an unpaired t-test with a significant p-value in the MTT assay when administering increasing doses of doxorubicin. The obtained chemosensitivity results indicated that chronic cyclic hypoxia plays an imperative role in the development of chemoresistant phenotypes in pancreatic and breast cancer cells; thus, understanding the differential expression of miRNA between hypoxic and normal cells may assist in the introduction of new markers to target.

It has been previously reported that combining oxaliplatin with a miR-181a-5p inhibitor enhances the apoptotic pathway in pancreatic cancer cells by targeting ATMs (13). In addition, the downregulation of let-7e enhances DNA repair by targeting PARP1 and thus induces cisplatin resistance in ovarian cancer (14). Based on the finding that miR-181 is consistently overexpressed in both 10 and 20 cycles of hypoxia, whereas let-7e is consistently downregulated, we can speculate that miRNA-181 and let-7e may be involved in pancreatic cancer cells’ resistance to doxorubicin under hypoxic conditions (15). MiR-17 and miR-93 were consistently downregulated in MCF7 miRNA profiling at the 10th and 20th hypoxia cycles, with a twofold decline in the fold change for each. According to Xia et al., the loss of miR-17 and miR-20b boosted breast cancer resistance to taxol by upregulating nuclear receptor coactivator 3 (NCOA3) levels (16). Furthermore, downregulated miR-93 induced chemoresistance to paclitaxel by targeting CCND1 and E2F1 (17). Moreover, miR-34a and miR-27 were both upregulated in cycle 10 and may participate in doxorubicin chemoresistance via targeting of the delta-like ligand 1 (DLL1) receptor, a Notch pathway ligand (18), and XIAP axis (19), respectively. Based on previous studies and their positive correlation with our miRNA expression profile, we may infer that these miRNAs have an important role in hypoxic MCF7 doxorubicin resistance.

The results of this study point to several miRNAs that may play a role in modulating chemotherapy response in pancreatic and breast cancer patients. Before these associations can be suggested as biomarkers of response to chemotherapeutic treatments in pancreatic and breast cancer, more research is required to uncover the molecular mechanisms underlying them.

### 4.1 An illustration of how differential miRNA expression affects cancer hallmarks

#### 4.1.1 PANC-1 enriched pathways

According to the hypoxic PANC-1 miRNA expression profile analysis, cell proliferation pathways were some of the most enriched pathways. Hypoxia reduces overall cell proliferation in most cell types, because an increased cell number, with a corresponding rise in oxygen demand, will only exacerbate hypoxia-induced stress (20). However, as the proliferation rate of hypoxic PANC-1 cells was comparable to that of normoxic cells, this assumption is irrelevant to our investigation. The deregulation of specific miRNAs could dramatically orchestrate cell proliferation after PANC-1 cells were exposed to chronic cyclic hypoxia. Previous studies have shown that miR-15b and miR-221 downregulation enhances cell proliferation through targeting FGF7, WNT3A (21), and eukaryotic translation initiation factor 5A2 (EIF5A2) (22), respectively. On the other hand, cell proliferation may be halted by the downregulation of miR-21 which targets the cell proliferation inhibitor, sprouty RTK signaling antagonist 2 (Spry2) (23), and the upregulation of miR-34 which targets CDK4 and c-Myc (24). Thus, the effect of these differentially expressed miRNAs on cell proliferation at the 10^th^ cycle of hypoxia is debatable. At the 20^th^ cycle of hypoxia, additional miRNAs that inhibit cell proliferation were downregulated, including miR-29b, miR-378, miR-155, and miR-7, which target CDK14 (25), BRAF (26), IGF2 (27), and SOX18 (28), respectively. Collectively these studies explained why, even after 20 cycles of hypoxia, the hypoxic PANC-1 proliferation rate was still comparable to its normoxic counterpart.

Another pathway that enriched significantly in the hypoxic PANC-1 miRNA expression profile was evading apoptosis. By targeting histone deacetylase-6 (HDAC6), a key player in the cellular control of protein aggregates, the downregulation of miR-221 suppresses autophagy (29). Moreover, downregulation of miR-101, miR-29, miR-7, miR-135, and miR-155, as well as the overexpression of miR-181a, may all have played a role in modulating PANC-1 cell death at cycle 20. MiR-181a has been shown to promote the development of pancreatic cancer by suppressing the tumor suppressor genes PTEN and MAP2K4 (30), whereas downregulated miR-155, miR-135a, miR-7, miR-29b, and miR-101 aid in apoptosis evasion by the targeting of X-linked inhibitor of apoptosis (XIAP) (31), Bmi1 (32), MAP3K9 (33), DNMT3b (3), and RLIP76 (34), respectively.

Our results contrast with some studies indicating that the induction of apoptosis is clearly linked to upregulated miR-34, which targets Bcl-2 and Notch (35), as well as downregulated let-7f, which targets caspase 3 and caspase 9 (36), and the intermittent hypoxia/re-oxygenation (IHR) downregulated miR-21, which targets TNF and EIF2 (37). This evidence suggests that hypoxic signaling does not exclusively contribute to oncogenic effects and could also be associated with tumor-suppressive and pro-apoptotic cascades.

Metastasis is a multi-step, dynamic biological process in which the EMT plays a critical role. Growing evidence shows that miRNAs have an important role in EMT and cancer metastasis (38). Regardless of miR-34 tumor suppressor activity via targeting Snail1 and Notch1 (39), other deregulated miRNAs suggest PANC-1 metastatic stimulation under 10 cycles of hypoxia. miR-21 downregulation increases Tiam1 expression which is attributed to cancer metastatic potential (15). Moreover, miR-155-null macrophages were susceptible to M2 polarization due to increased expression of C/EBPβ, a recognized miR-155 target, thus modulating solid tumor metastasis (40).

The downregulation of miR-29 and miR-378 could have played important roles in fostering metastasis, at cycle 20, by targeting matrix metalloproteinase 2 (MMP2) (41) and vimentin (42), respectively. Depending on previous studies and our findings, which indicate an evident morphological transformation in hypoxic PANC-1 cells from epithelial to mesenchymal phenotype with consistent metastasis related-miRNA deregulations, we may deduce that chronic cyclic hypoxia promotes pancreatic cancer metastasis. Angiogenesis is a well-coordinated tumor growth and metastasis process that entails the creation of new blood vessels from pre-existing ones to satisfy the cell’s needs (38). The pathways that related to angiogenesis were exclusively enriched in the 10^th^ cycle miRNA expression profile. Intriguingly, the effect of miR-21-5p on its target, SMAD7, expression was markedly different between normoxia and hypoxia; in normoxia, the transfection of miR-21-5p mimicked enhanced tube-forming ability, but in hypoxia, it diminished. In addition, inhibiting miR-21 improved blood flow in a skin flap, indicating that miR-21 inhibits angiogenesis in human endothelial cells and rat skin flaps under hypoxic conditions (43). miR-221-3p downregulation, moreover, induce angiogenesis via targeting HIF-1 (44). In contrast, Gnanamony et al. reported that the circular dumbbell miR34a -3p and -5p suppresses angiogenesis in pancreatic cancer cells (45).

The DNA damage response (DDR) pathway was another enriched pathway. Surprisingly, the DDR mechanism is involved in both the development and treatment of cancer. A rising number of miRNAs have been found to intervene in processes regulating tumorigenesis and responses to radiation or chemotherapies by modifying the DDR (46). A study showed that the exogenous addition of miR-34 affects breast cancer cell survival after radiotherapy and suggests that anti-miR-34 may be used as a radiosensitizing agent in p53-mutant breast cancer (47). In addition, DNA repair factors are targeted by several miRNAs, including miR-155 which targets RAD51, MLH1, and MSH6 (48), miR-15b which targets the inhibitor of nuclear factor kappa B kinase subunit beta (IKBKB) and WEE1 (49), and miR-101 which targets RRM1 (50). These studies support what was reported in the miRNA expression profile that the upregulation of miR-34a, as well as the downregulation of miR-15b, miR-155, and miR-101, might improve DNA repair and hence contribute to cancer cell survival and chemoresistance.

At the 10^th^ cycle, the downregulation of miR-155, miR-221, and let-7f, and the upregulation of miR-34a could boost hypoxic PANC-1 inflammation and promote its chemoresistance. miR-34 pro-inflammatory functioning is mediated by LGR4 (51). The downregulation of the inflammation-associated miRNA, miR-155, promotes M2 macrophages, which play a key role in the immunosuppressive microenvironment. Furthermore, let-7f and miR-221 downregulation promote inflammation by targeting NLRP3 (52), p38/NF-Kβ, and ICAM-1 (53), respectively. The downregulation of miR-101 which targets COX2 (54), miR-135a which targets toll-like receptor 4 (TLR4), and miR-7 (55), as well as the upregulation of miR-181a which is linked to IL-1, IL-6, and TNFα expression (56), all may contribute to inflammation potentiation at cycle 20. The majority of previous studies support the development of the PANC-1 inflammatory response by chronic cyclic hypoxia.

#### 4.1.2 MCF7 enriched pathways

In the case of MCF7, chronic cyclic hypoxia had a significant impact on four cancer pathways: cell proliferation, apoptosis, angiogenesis, and DDR. The miRNA expression profile of hypoxic MCF7 cells was enriched for cell proliferation pathways, and several tumor suppressor miRNAs that target cell proliferation regulators were downregulated at the 10^th^ cycle, including miR-17-5p that targets AIB1 and insulin-like growth factor 1 (57), miR-20a that targets MAPK1 (58), miR-205 that targets E2F1 (59), and miR-15a that targets cyclin E1 (CCNE1) (60). Moreover, the upregulation of miR-27a induces cell proliferation via the upregulation of Wnt-β-catenin and SFRP1 targeting (61). In contrast, miR-34a inhibits BC proliferation, invasion, and migration by deactivating the Wnt-β-catenin signaling pathway (62), raising the question of whether hypoxia has a negative or positive effect on the Wnt-β-catenin signaling pathway.

Other downregulated tumor suppressor miRNAs may have potentiated MCF7 proliferation at cycle 20. Downregulated let-7a-5p induces glycolysis via PKM2 and GLUT12 targeting (63, 64). Furthermore, reduced miR-16-5p, miR-9-5p, and miR-195 enhanced breast cancer cell proliferation, and accelerated the cell cycle by targeting AKT3 (65), androgen receptor signaling (66), and cyclin E1 (CCNE1) (67), respectively. The emergence of evading apoptosis hallmarks was expected to become evident at cycle 20, in line with the behavior of breast cancer cells from the 12th to the 15th cycle of hypoxia, where the proliferation rate decreased and the apoptosis rate increased significantly, and then the cells returned to grow and multiply, defeating apoptosis. Several tumor suppressor miRNAs, whose upregulation has been linked to apoptosis in previous studies, were downregulated in this study. For instance, ectopic expression of miR-15a and miR-16 induced mitochondrial reactive oxygen species, followed by cytochrome-C release into the cytosol, which triggered caspase-3 and caspase-6/9, resulting in intrinsic apoptosis. Additionally, BMI1 and BCL2 were significantly downregulated by miR-15a and miR-16 (68). miR-9-5p, miR-195, miR-7-5p, and miR-101 also exhibit pro-apoptotic activity via the targeting of MTHFD2 (69), Bcl-2 (70), proteasome activator subunit 3 (REGγ) (71), and JAK2 (72), respectively.

Increased tumor angiogenesis and upregulated expression of the proangiogenic factor VEGF-A contribute to the tumorigenicity of breast cancer cells as a result of EMT (73). Hypoxic MCF7 exhibited the downregulation of many anti-angiogenic miRNAs at cycle 10, including miR-15a/16 which targets VEGF-A (74), miR-17 which targets several tumor angiogenesis-inducing genes, including TGFBR2, HIF1, and VEGFA (75), miR-93 which targets WNK lysine deficient protein kinase 1 (WNK1) (76), and miR-20a which targets VEGF (77). The miR-205/YAP1 signaling pathway, in particular, converts normal breast fibroblasts to CAFs. Angiogenesis and therefore breast cancer cell invasion and metastasis are inhibited when miR-205 is restored in CAFs (78). ErbB3 and VEGF-A are considered direct targets for miR-205 (79). miR-34a, on the other hand, inhibits epithelial cell-mediated angiogenesis by inducing senescence through Sirt1 (80). Additionally, in terms of miRNA expression at cycle 20, downregulation of miR-16-5p targets VEGFA (81). All of this evidence is consistent with our differential miRNA expression findings, which affirm angiogenesis as a reasonable response to chronic cyclic hypoxia that breast cancer cells are exposed to, as well as reinforce the angiogenesis-EMT relationship, which fuels breast cancer metastasis.

The DDR pathway was significantly enriched in the hypoxic MCF7 miRNA expression profile. Low levels of miR-15a and miR-16 desensitize breast cancer cells to doxorubicin, resulting in less apoptotic cell death by targeting the DNA repair factor BMI1 (82). As previously mentioned, miRNA-34a facilitates DNA damage repair, too. Furthermore, miR-27a targets ATM and facilitates cell proliferation even after irradiation (83). At cycle 20, miR-15b and miR-16, in addition to miR-15a, played a role in DDR. miR-15b targets Wip1 and induces the DNA damage response (84). Collectively, these studies show an increase in DNA repair by chronic cyclic hypoxia and thus the development of resistant and aggressive cancer cells.

As expected, the miRNA expression profiles of PANC-1 and MCF7, after chronic cyclic hypoxia, deregulated differently; this can be explained by the fact that the two cell lines tolerate hypoxia in distinct manners. The pancreatic cancer cell line studied in the present study may be particularly tolerant of hypoxia since it survives oxygen levels 25 times lower than its normal counterpart. Whereas, the breast cancer cell line studied is considered less hypoxic since it survives oxygen levels 5 times less than normal breast cancer tissue (85).

## 5 Conclusions

According to this study, distinct patterns of dysregulated miRNA expression in PANC-1 and MCF7 cell lines under chronic cyclic hypoxia could aid in highlighting novel hypoxamiRs to target. During the 20 cycles of pancreatic and breast cancer hypoxia, four different miRNAs appeared to be key mediators of various cancer hallmarks and may have great potential as novel targets. miR-221, miR-21, miR-155, and miR-34 could orchestrate proliferation, apoptosis, DDR, metastasis, angiogenesis, and inflammation of hypoxic PANC-1, whereas miR-93, miR-20a, miR-17, and miR-15 could regulate proliferation, apoptosis, angiogenesis, and DDR of hypoxic MCF-7 cells. Furthermore, differential expression of miR-181a and let-7e in PANC-1, as well as miR-93, miR-34, and miR-27 in MCF7 may be linked to the development of chemoresistant MCF7 and PANC-1 cells after 20 cycles of chronic cyclic hypoxia. The particular mechanisms that govern the aforementioned processes are yet unknown; thus, the interpretation of miRNA profiling data and the regulatory functions of miRNA in cancer development and progression should be further investigated.

## Data availability statement

The datasets presented in this study can be found in online repositories. The names of the repository/repositories and accession number(s) can be found in the article/**Supplementary Material**.

## Author contributions

SA-S conceptualized and conducted the experiments, carried them out, analyzed the data, constructed figures and/or tables, and wrote the manuscript. MZ conceptualized and conducted the experiments, analyzed the data, constructed figures and/or tables, and reviewed versions of the manuscript. HH conceptualized the experiments and undertook funding acquisition. All authors contributed to the article and approved the submitted version.

## Glossary

**Table:**

AIB1	amplified in breast cancer 1
AKT3	AKT serine/threonine kinase 3
ATM	ataxia-telangiectasia mutated
BC	breast cancer
Bcl-2	B-cell lymphoma 2
Bmi1	B cell-specific Moloney murine leukemia virus integration site 1
C/EBPβ	CCAAT-enhancer-binding proteins beta
CAFs	cancer-associated fibroblast
CCND1	cyclin D1
CDK	cyclin-dependent kinase
COX2	cyclooxygenase 2
DDR	DNA damage response
DLL1	delta like ligand 1
DNMT3b	DNA methyltransferase 3 beta
E2F1	E2 promoter binding factor 1
EIF5A2	eukaryotic translation initiation factor 5A2
EMT	epithelial-to-mesenchymal transition
ErbB3	erb-B2 receptor tyrosine kinase 3
FGF7	fibroblast growth factor 7
GLUT12	glucose transporter 12
HDAC6	histone deacetylase-6
HIF1	hypoxia-inducible factor
ICAM-1	intercellular adhesion molecule 1
IGF2	insulin like growth factor 2
IKBKB	inhibitor of nuclear factor kappa B kinase subunit beta
IL	interleukin
JAK2	Janus kinase 2
MAPK1	mitogen-activated protein kinase 1
miRNA	microRNAs
MLH1	MutL homolog 1
MMP2	matrix metalloproteinase 2
MSH6	MutS homolog 6
MTHFD2	mitochondrial methylene tetrahydrofolate dehydrogenase
MTT	3-(4, 5-dimethylthiazol-2-yl)-2,5-diphenyl tetrazolium bromide
NCOA3	nuclear receptor coactivator 3
NF-Kβ	nuclear factor kappa-light-chain-enhancer of activated B cells
PARP1	poly (ADP-ribose) polymerase 1
PDAC	pancreatic ductal adenocarcinoma
PKM2	pyruvate kinase isoenzyme type M2
PTEN	phosphate and tensin homolog
RAD51	RAD51 homolog, DNA repair protein
RLIP76	Ral interacting protein of 76 kDa
RT-PCR	real time polymerase chain reaction
SFRP1	secreted frizzled-related protein 1
Sirt1	sirtuin (silent mating type information regulation 2 homolog) 1
Snail1	zinc finger protein
SOX18	SRY-box transcription factor 18
Spry2	sprouty RTK signaling antagonist 2
TGFBR2	transforming growth factor, beta receptor II
TLR4	toll-like receptor 4
TME	tumor microenvironment
TNF	tumor necrosis factor
VEGF-A	vascular endothelial growth factor A
WEE1	WEE1 G2 checkpoint kinase
Wip1	wild-type p53-induced phosphatase 1
WNK1	WNK lysine deficient protein kinase 1
WNT3A	Wnt family member 3A
XIAP	X-linked inhibitor of apoptosis
