# Supplementary material for: Differential miRNA expression of hypoxic MCF7 and PANC-1 cells
Source: Front Endocrinol (Lausanne). 2023 Jul 31;14:1110743. doi: 10.3389/fendo.2023.1110743 (PMC10424510; doi:10.3389/fendo.2023.1110743)
Supplement: Supplementary file 1 [file Table_1.docx]

**Appendix A. Supplementary data**

- Table 1. Concentration and purity of RNA samples

| **Cells** | **Cycle Number** | **RNA concentration ng/µl** | **RNA purity 260/280** |
| --- | --- | --- | --- |
| PANC-1 | 10^th^ | 1620.6 | 2.07 |
| PANC-1 | 10^th^ | 1547.1 | 2.05 |
| PANC-1 | 20^th^ | 1394.3 | 2.04 |
| PANC-1 | 20^th^ | 1364.8 | 2.05 |
| PANC-1 | Normoxia | 1811.1 | 2.06 |
| PANC-1 | Normoxia | 1881.9 | 2.05 |
| MCF7 | 10^th^ | 1247.7 | 2.06 |
| MCF7 | 10^th^ | 1186.2 | 2.06 |
| MCF7 | 20^th^ | 245.6 | 2.02 |
| MCF7 | 20^th^ | 227.3 | 1.96 |
| MCF7 | Normoxia | 600.5 | 2.06 |
| MCF7 | Normoxia | 468.8 | 2.02 |

- **PCR array-based miRNA transcriptomic profiling of PANC-1**

Table 2. Significantly altered miRNAs of hypoxic vs normoxic PANC-1 after 10 cycles of hypoxia

| **miRNA Symbol** | **Fold change** |
| --- | --- |
| hsa-miR-429 | -30.132 |
| hsa-miR-7-5p | -10.821 |
| hsa-miR-378a-3p | -6.3788 |
| hsa-miR-10b-5p | -4.7291 |
| hsa-miR-191-5p | -4.3767 |
| hsa-miR-877-3p | -4.0846 |
| hsa-let-7f-5p | -4.0676 |
| hsa-miR-15b-5p | -3.607 |
| hsa-miR-98-5p | -3.3675 |
| hsa-miR-155-5p | -3.0619 |
| hsa-let-7e-5p | -2.6704 |
| hsa-miR-935 | -2.5004 |
| hsa-miR-99a-5p | -2.4036 |
| hsa-miR-221-3p | -2.389 |
| hsa-miR-21-5p | -2.366 |
| hsa-miR-23a-3p | -2.3394 |
| hsa-miR-204-5p | -2.3277 |
| hsa-miR-9-5p | -2.321 |
| hsa-miR-491-5p | -2.1176 |
| hsa-miR-125a-5p | -2.0164 |
| hsa-miR-22-3p | 2.3673 |
| hsa-miR-181c-5p | 2.9597 |
| hsa-miR-210-3p | 3.5891 |
| hsa-miR-181a-5p | 3.9413 |
| hsa-miR-34a-5p | 4.5799 |

Table 3. Significantly altered miRNAs of hypoxic vs normoxic PANC-1 after 20 cycles of hypoxia

| **miRNA Symbol** | **Fold change** |
| --- | --- |
| hsa-miR-429 | -36.869 |
| hsa-miR-7-5p | -5.717 |
| hsa-miR-155-5p | -4.1415 |
| hsa-miR-146b-5p | -4.0546 |
| hsa-miR-101-3p | -3.4783 |
| hsa-miR-135a-5p | -3.4289 |
| hsa-miR-449a | -3.3341 |
| hsa-miR-122-5p | -3.0912 |
| hsa-miR-191-5p | -3.0852 |
| hsa-miR-378a-3p | -2.7188 |
| hsa-miR-935 | -2.3581 |
| hsa-miR-99a-5p | -2.3301 |
| hsa-miR-491-5p | -2.3132 |
| hsa-miR-29b-3p | -2.3059 |
| hsa-let-7e-5p | -2.2885 |
| hsa-miR-21-5p | -2.1917 |
| hsa-miR-188-5p | -2.1087 |
| hsa-miR-221-3p | -2.0319 |
| hsa-miR-210-3p | 2.3054 |
| hsa-miR-181c-5p | 2.7858 |
| hsa-miR-181a-5p | 2.9116 |
| 0hsa-miR-335-5p | 3.2233 |
| hsa-miR-324-5p | 3.6049 |
| hsa-miR-34a-5p | 6.0687 |

- **PCR array-based miRNA transcriptomic profiling of MCF7**

Table 4. Significantly altered miRNAs of Hypoxic Vs Normoxic MCF7 after 10 cycles of hypoxia

| **miRNA Symbol** | **Fold change** |
| --- | --- |
| hsa-miR-99a-5p | -19.911 |
| hsa-miR-188-5p | -8.3403 |
| hsa-miR-125b-5p | -5.0646 |
| hsa-miR-449a | -3.8686 |
| hsa-miR-15a-5p | -3.3759 |
| hsa-miR-205-5p | -3.3347 |
| hsa-miR-378a-3p | -2.7095 |
| hsa-miR-17-5p | -2.6435 |
| hsa-miR-20b-5p | -2.6261 |
| hsa-miR-19a-3p | -2.6074 |
| hsa-miR-20a-5p | -2.5648 |
| hsa-miR-93-5p | -2.4855 |
| hsa-miR-101-3p | -2.3689 |
| hsa-miR-125a-5p | 2.047 |
| hsa-miR-215-5p | 2.1098 |
| hsa-miR-27a-3p | 2.3356 |
| hsa-miR-34a-5p | 2.3543 |
| hsa-miR-23a-3p | 2.4945 |
| hsa-miR-29b-3p | 2.8036 |

Table 5. Significantly altered miRNAs of Hypoxic Vs Normoxic MCF7 after 20 cycles of hypoxia

| **miRNA Symbol** | **Fold change** |
| --- | --- |
| hsa-miR-99a-5p | -21.01 |
| hsa-miR-188-5p | -13.093 |
| hsa-miR-378a-3p | -9.688 |
| hsa-miR-125b-5p | -9.1267 |
| hsa-miR-449a | -6.9741 |
| hsa-miR-195-5p | -6.1639 |
| hsa-miR-20a-5p | -5.7934 |
| hsa-miR-15b-5p | -5.6219 |
| hsa-miR-16-5p | -5.3026 |
| hsa-miR-205-5p | -5.1405 |
| hsa-miR-17-5p | -5.0454 |
| hsa-miR-101-3p | -4.9598 |
| hsa-miR-20b-5p | -4.7035 |
| hsa-miR-93-5p | -4.6331 |
| hsa-miR-7-5p | -4.4978 |
| hsa-miR-192-5p | -4.1739 |
| hsa-miR-92a-3p | -4.169 |
| hsa-miR-324-5p | -4.158 |
| hsa-miR-19a-3p | -4.089 |
| hsa-miR-130b-3p | -3.7284 |
| hsa-miR-186-5p | -3.6953 |
| hsa-let-7c-5p | -3.3867 |
| hsa-let-7d-5p | -3.2257 |
| hsa-miR-146b-5p | -3.2017 |
| hsa-miR-429 | -3.1594 |
| hsa-let-7i-5p | -2.9706 |
| hsa-miR-23b-3p | -2.8899 |
| hsa-miR-191-5p | -2.849 |
| hsa-miR-98-5p | -2.7561 |
| hsa-miR-148b-3p | -2.7559 |
| hsa-miR-9-5p | -2.7556 |
| hsa-miR-877-3p | -2.7235 |
| hsa-miR-148a-3p | -2.7184 |
| hsa-miR-103a-3p | -2.718 |
| hsa-miR-184 | -2.4062 |
| hsa-miR-15a-5p | -2.3688 |
| hsa-miR-26a-5p | -2.3485 |
| hsa-miR-135a-5p | -2.28 |
| hsa-let-7a-5p | -2.2752 |
| hsa-miR-181b-5p | -2.2491 |
| hsa-miR-30e-5p | -2.2333 |
| hsa-miR-200b-3p | -2.0876 |
| hsa-let-7b-5p | -2.0717 |
| hsa-miR-203a-3p | -2.0563 |
| hsa-let-7f-5p | -2.0299 |
| hsa-miR-215-5p | 2.517 |
| hsa-miR-29b-3p | 2.9424 |
